# Second language social networks and communication-related acculturative stress: the role of interconnectedness

**DOI:** 10.3389/fpsyg.2015.01111

**Published:** 2015-08-04

**Authors:** Marina M. Doucerain, Raheleh S. Varnaamkhaasti, Norman Segalowitz, Andrew G. Ryder

**Affiliations:** ^1^Department of Psychology, Concordia University, Montreal, QCCanada; ^2^School of Psychology and Counselling, Faculty of Health, Queensland University of Technology, Brisbane, QLDAustralia; ^3^Culture and Mental Health Research Unit, Lady Davis Institute, Jewish General Hospital, Montreal, QCCanada

**Keywords:** social networks, acculturation, acculturative stress, intercultural communication, cultural adaptation

## Abstract

Although a substantial amount of cross-cultural psychology research has investigated acculturative stress in general, little attention has been devoted specifically to communication-related acculturative stress (CRAS). In line with the view that cross-cultural adaptation and second language (L2) learning are social and interpersonal phenomena, the present study examines the hypothesis that migrants’ L2 social network size and interconnectedness predict CRAS. The main idea underlying this hypothesis is that L2 social networks play an important role in fostering social and cultural aspects of communicative competence. Specifically, higher interconnectedness may reflect greater access to unmodified natural cultural representations and L2 communication practices, thus fostering communicative competence through observational learning. As such, structural aspects of migrants’ L2 social networks may be protective against acculturative stress arising from chronic communication difficulties. Results from a study of first generation migrant students (*N* = 100) support this idea by showing that both inclusiveness and density of the participants’ L2 network account for unique variance in CRAS but not in general acculturative stress. These results support the idea that research on cross-cultural adaptation would benefit from disentangling the various facets of acculturative stress and that the structure of migrants’ L2 network matters for language related outcomes. Finally, this study contributes to an emerging body of work that attempts to integrate cultural/cross-cultural research on acculturation and research on intercultural communication and second language learning.

## Introduction

Don tilted his beetle eyebrows and asked, ‘Tell me, why did you leave that place?’

‘My bawss was sacked, so we got laid all together.’

‘You got what?’ Don asked with a start. A young secretary at another desk tittered.

(Jin, 2007 , p. 25)

In this excerpt of Ha Jin’s novel *A Free Life*, Nan, a Chinese immigrant in the US startled his interlocutor during a job interview by inadvertently omitting the preposition ‘off’ of the phrasal verb ‘to lay off.’ This kind of communication breakdown, as well as other types of difficulty arising from varying cultural norms surrounding communication practices, is a common experience for migrants. In the example above, Nan was able to repair the conversation and eventually obtained the job he was seeking; such happy outcomes are by no means guaranteed, however, and chronically experiencing communication difficulties can be stressful for migrants (Kang, 2006). Yet, although a substantial amount of cross-cultural psychology research has investigated acculturative stress in general, little attention has been devoted specifically to communication-related acculturative stress (CRAS). This research gap is unfortunate, as communication-related stress may impact not only migrants’ well-being but also important aspects of second language (L2) learning such as their willingness to communicate with L2 speakers (MacIntyre et al., 1998). As such, CRAS can have negative implications for migrants’ social integration into the mainstream community.

In line with current perspectives that view intercultural communication as a key mechanism underlying cultural adaptation (Kim, 2001), the present study examines the hypothesis that the size and structure of migrants’ L2 social networks are important predictors of CRAS. Given that migrants’ ability to communicate in the dominant language of the new cultural environment (that is, in an L2) is a core aspect of cross-cultural adaptation, a better understanding of the antecedents of CRAS is essential. Despite Smith’s (1999) argument that social networks are ideally suited to research on cross-cultural adaptation, this approach has received surprisingly little empirical attention in areas related to acculturation and intercultural communication. The present study seeks to address this gap, as well as to integrate cross-cultural research on acculturation and research on L2 learning and intercultural communication.

## Acculturation, Language, and Stress

### The Role of Language in Acculturation

Psychological acculturation refers to the changes experienced by a person as a result of continuous first-hand cross-cultural contact, as s/he strives to be functional in the cultural contexts relevant to her/him (Kim, 2001; Berry, 2005). In the case of migrants, these changes are typically far reaching and lead to an extensive reconfiguration of their lives – beyond acquiring a new language, understanding new cultural traditions, and learning new social norms, migrants need to form new social relationships, as well as create new and/or adjust old identities (Sam and Berry, 2010). To a large extent, these transformations occur through social interactions in the new environment. Migrants acquire knowledge of a new cultural tradition and negotiate their social position in the new environment through repeated communication activities, be it with members of the new cultural group or with cultural artifacts (e.g., television programs, advertisements, internet pages). As such, it is unsurprising that language and L2 competencies occupy a key position in most accounts of acculturation, both in the field of cross-cultural psychology (Noels et al., 1996; Masgoret and Ward, 2006) and of intercultural communication (Nishida, 1999; Kim, 2001; Gudykunst, 2005). Thus, the theoretical perspective adopted here views processes of cross-cultural adaptation as occurring “in and through communication” (Kim, 2001, p. 36). While successful communication serves migrants’ goals and reflects an adaptive level of social functioning (Gallagher, 2013), intercultural communication difficulties can potentially hinder cross-cultural adaptation.

### Acculturative Stress

Stemming from a stress and coping perspective (Lazarus and Folkman, 1984), most research on psychological acculturation has examined the well-being and adjustment consequences of acculturative changes. Supporting the importance of language in acculturation, a number of studies showed that L2 competencies are a key predictor of adjustment (e.g., Noels et al., 1996; Kim, 2005; Vedder and Virta, 2005; Kang, 2006). The construct of acculturative stress, referring to “a stress reaction in response to life events that are rooted in the experience of acculturation” (Sam and Berry, 2010, p. 474), lies at the core of this research on migrants’ well-being. Acculturative stress arises in situations where acculturative pressures exceed migrants’ perceived ability to cope. Studies have found associations between acculturative stress and a range of negative outcomes, such as depression, suicide ideation, alcohol abuse, and self-reported physical health (Gil et al., 1994; Hovey and King, 1996; Finch et al., 2001).

There is little doubt that many aspects of cross-cultural adaptation can be stressful, but critics have suggested that acculturative stress has come to represent a “catch-all concept for every kind of problem that minorities might encounter,” thus resulting in “a history of confusion and confounds” (Rudmin, 2009, p. 116). Indeed, accounts of acculturative stress, as well as scales measuring the construct, typically encompass a variety of difficulties, ranging from discrimination issues to communication difficulties to cultural isolation (e.g., Rodriguez et al., 2002; Benet-Martínez and Haritatos, 2005). We agree with Rudmin’s (2009) critique – acculturative stress is a “catch-all” concept – and believe that it might be important to examine classes of stressors separately. The antecedents and consequences of perceived cultural incompatibility may be quite different from those related to, say, work difficulties. To date, very little work has focused on “unpacking” acculturative stress. As a notable exception, Benet-Martínez and Haritatos (2005) examined the personality antecedents of different aspects of acculturative stress as well as the differential ability of these aspects to predict bicultural identity integration.

In line with this view on the importance of unpacking acculturative stress, we focus specifically on CRAS. Conceptualizations of acculturative stress vary in the types of difficulties they encompass, but they consistently include L2 and intercultural communication issues. In fact, most commonly used acculturative stress scales contain items addressing language and communication difficulties (e.g., Social Attitudinal Familial and Environmental Acculturative Stress Scale: Padilla et al., 1985; Acculturative Stress Scale for International Students: Sandhu and Asrabadi, 1994; Multidimensional Acculturative Stress Inventory: Rodriguez et al., 2002; Riverside Acculturative Stress Index: Benet-Martínez and Haritatos, 2005).

### Communication-Related Acculturative Stress

Communication-related acculturative stress is defined here as migrants’ subjective stress reaction in response to chronic difficulties in L2-mediated communication with members of the mainstream cultural group. Both limited linguistic knowledge (i.e., knowledge of syntax, morphology, lexicon, and phonology of a language) and limited competence in sociocultural or pragmatic aspects of communication can lead to intercultural communication breakdowns (Thomas, 1983) and thus result in feelings of incomprehension and frustration, both in native and non-native interlocutors.

We conceptualize CRAS as migrants’ reaction to the regular occurrence of such situations. In turn, this stress reaction may impact not only migrants’ well-being but also L2 learning variables such as willingness to communicate (MacIntyre et al., 1998) or anxiety and uncertainty in the face of intercultural communication events (Gudykunst, 2005). As such, CRAS may provide a link for integrating research in cross-cultural psychology on culture acquisition and adjustment with research on intercultural communication and on second language acquisition (SLA). With this work, we seek to contribute to a growing body of work (e.g., Noels et al., 1996; Gallagher, 2013) that aims to integrate these relatively separate strands of research. Our main goal is to examine the role of migrants’ L2 social network size and structure in predicting CRAS.

## Communication-Related Acculturative Stress and L2 Social Networks

The re-creation of a social architecture in a new cultural environment is a central task of acculturation (Kuo and Tsai, 1986). It allows migrants to re-establish an adequate support system and to gain access to resources that will facilitate cross-cultural adaptation. In particular, migrants’ L2 social networks, referring to social relationships mediated through the L2, are critical for acquiring knowledge of the new cultural tradition (Smith, 1999) and for fostering communicative competence in the L2 (Cenoz and Valencia, 1993; Smith, 2002). Similarly, Kim (2001, p. 123) argues that through engagement in their L2 social network, migrants can, “confirm or reject presumed meanings and motives in natives’ communication behaviors.” Furthermore, migrants’ L2 social networks “exert social control by determining the language [migrants] must use and by conveying messages of cultural values and social approval or disapproval” (p. 123). This argument not only underscores the potential importance of L2 social networks in cross-cultural adaptation but also points to their specific role in facilitating socio-cultural and pragmatic aspects of intercultural communication.

Past research (reviewed extensively in Gallagher, 2012) has examined the relation between competence in L2 and L2 social networks. Cenoz and Valencia (1993) found that among Spanish first language (L1) participants, proficiency in Basque L2 was associated with the proportion of Basque speakers in their social network. In a study of immigrant students to Sweden, Wiklund (2002) detected a similar relation between L2 Swedish proficiency and more Swedish-oriented social networks, although her results were limited to a description of proportions. Similarly, in a case study, Smith (2002) found a positive association between communicative competence and proportion of members of the mainstream cultural group in the social network of migrants. In parallel, several studies have examined the relation between social networks and adjustment. Kuo and Tsai (1986) found that higher interconnectedness among closest friends of Asian migrants to the US was related to less depression. Similarly, García et al. (2002) showed that migrant women in Spain who included more Spaniards in their social networks experienced less depression. Taken together, these sets of results support the general hypothesis that characteristics of migrants’ L2 social networks may predict CRAS. More specifically, we expect a negative relation between L2 social network size and CRAS.

These studies have focused primarily on the role of the number of social ties in predicting outcomes of interest. Conceptually, however, social network theory emphasizes that people are embedded in webs of social relations (Borgatti et al., 2009) and that the *structure* of the system influences and place constraints on individual actors within it. In line with this perspective, egocentric network analysis (analyzing an individual’s personal network in contrast to analyzing a complete bounded network such as a school class or a department in a corporation) is primarily concerned with how characteristics of the social structure within which a person is embedded are associated with outcomes of interest for that person (Carolan, 2014) – here, CRAS. The interconnectedness of a network – how tightly woven it is – is a commonly examined structural feature. It is positively associated with social support, and more interconnected networks facilitate the transmission of information and resources (Kadushin, 2012). Translated into language terms for the present case, this suggests that greater interconnectedness in migrants’ L2 social network may facilitate the transmission of normative language forms and communicative practices, and therefore be associated with lower CRAS. In a similar vein, Coleman (1988) argues that a tightly connected network fosters norm conformity. This is likely to be beneficial for migrants, as such networks afford greater exposure to a unified representation of cultural norms and L2 communication practices and promotes reinforcement of those norms and practices through various interconnected channels. As such, a tighter L2 social network has the potential to scaffold and regulate intercultural communication more closely. Furthermore, interconnectedness fosters trust and beneficial interdependence among network members (Coleman, 1988; Kadushin, 2012), which may also have positive consequences for intercultural communication. Indeed, Gudykunst (2005) argues that reduced anxiety/uncertainty is key to successful intercultural communication, and a network structure that favors trust would likely contribute to reducing communication anxiety and stress within that network.

In addition, greater interconnectedness among network members indexes a higher likelihood of taking part in triadic communication (since more network members know one another) and therefore of observing L2-mediated interactions between two members of the mainstream group. Thus, greater within-network interconnectedness would reflect greater access to natural cultural representations and L2 communication practices. That is, mainstream members could be expected to avoid adjusting or modifying their interactions with one another given their assumed shared cultural reality and their shared language. By contrast, during one-on-one interactions between a migrant and a mainstream member, the mainstream member might consciously or unconsciously tailor/adapt his or her discourse and communication practices to accommodate the migrant’s assumed cultural and communicative competence level. This effect, whereby the native speaker makes conversational adjustments to compensate for the interlocutor’s (perceived) linguistic deficits, has been well-documented in the SLA literature (see Wagner, 1996, for a review). In triadic interactions involving the migrant and two or more native speakers – which are more likely in more interconnected L2 networks – conversational adjustments by well-meaning native speakers are much less likely to occur. In short, cultural transmission of sociolinguistic competency skills might be facilitated in more interconnected networks through observational learning. In line with this hypothesis, recent work has shown that norms can emerge within a network through observational rather than direct learning (Kashima et al., 2013).

The above discussion underscores the positive potential of interconnected networks. In contrast, Burt (1995) contends that a tight network structure limits members’ access to new information and constrains their social roles and opportunities to explore new ideas. He proposes that the ability to bridge holes in the social structure (weaker connections between densely connected clusters) creates a competitive advantage and gives access to diversified information and resources. Burt’s (1995) formulation, however, makes no specific reference to the particularities of immigration and L2 communication contexts, which are typically characterized by social isolation (see, e.g., Williams and Johnson, 2011). In the present case, we would expect the benefits of an interconnected L2 social network to outweigh the detrimental constraints described by Burt (1995). Therefore, we expect a greater L2 network interconnectedness to be associated with lower CRAS.

**FIGURE 1:**
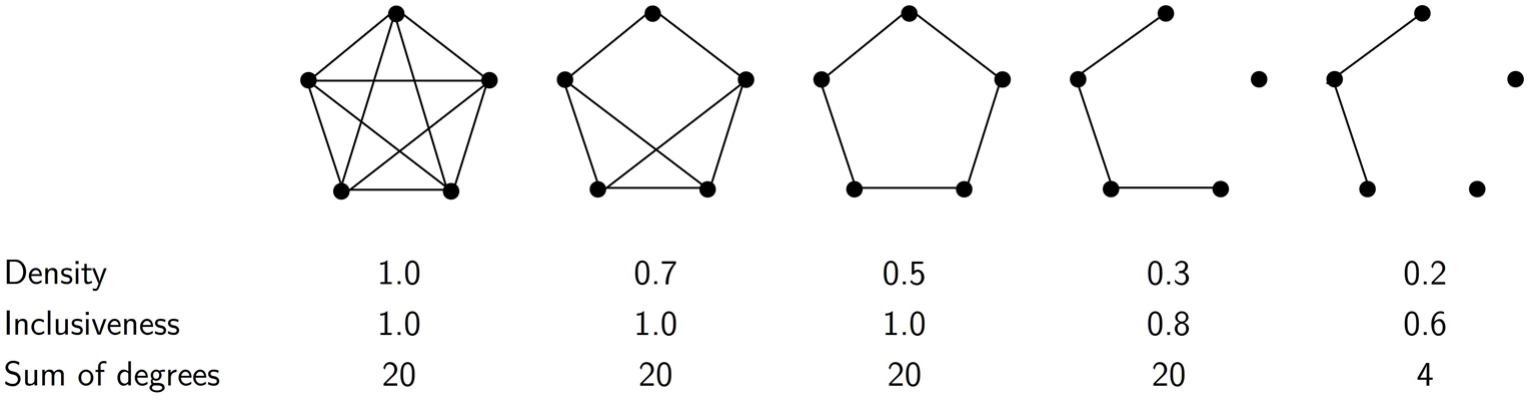
**Relation between density and inclusiveness of social networks (figure design inspired by Scott, 2012, p. 71)**.

Density is the most commonly used index of network interconnectedness (Scott, 2012). It is defined as the ratio between the number of existing connections among network members and the total number of potential connections within the network. Density is a function of two other structural parameters of a network (Scott, 2012): (1) the sum of the degrees of network members (the degree of a member is the number of connections the member has to other members) and (2) inclusiveness, or the proportion of network members who know at least one other person in the network. As such, both inclusiveness and density index the level of interrelatedness within a network but at different levels of granularity. As can be seen in **Figure 1**, higher inclusiveness entails a lower threshold of interrelatedness (knowing only one or ten other network members contributes equally to inclusiveness) than does density, where the extent to which each person is him/herself interconnected is taken into account. We expect both indices to be associated with CRAS, but we form no specific hypothesis regarding the relative strength of these associations.

## Specificity Considerations

In order to show that L2 social network size and structure uniquely predict variance in CRAS, we considered several alternative predictors. In addition to including sex and number of years lived in the country of settlement as demographic controls, we controlled for acculturation orientations, self-reported language proficiency, and overall intimacy in the L2 social network.

### Acculturation Orientations

Defined here as migrants’ motivation for cultural engagement and appreciation of a cultural tradition, acculturation orientations are arguably the most investigated antecedent of cross-cultural adaptation outcomes, with the general finding that more positive orientations toward both mainstream and heritage cultural groups are associated with better adjustment (see Nguyen and Benet-Martínez, 2013, for a meta-analysis). Past research also established a positive link between ethnolinguistic affiliation, language attitudes, and cultural attitudes on one side, and L2 competence and use on the other side (Moyer, 2007; Segalowitz et al., 2009; Gatbonton et al., 2011). Similarly, intergroup attitudes and intergroup motivation are considered to be an important antecedent of willingness to communicate, which plays a key role in L2 learning and intercultural communication. Acculturation orientations are undergirded by a suite of behavioral, cognitive, and affective mechanisms that facilitate an approach-oriented stance toward a given culture. As such, they are conceptually very close to variables such as ethnolinguistic affiliation or intergroup attitudes and motivation. For this reason, and for the sake of parsimony, we use acculturation orientation toward the mainstream cultural group as a proxy for the intra-individual affective–cognitive context motivating migrants’ L2 communication and engagement in the new cultural group, and acculturation orientation toward the heritage group as a proxy for affiliation with the heritage ethnolinguistic group.

### Self-Reported L2 Proficiency

The main idea underlying the hypothesized relation between social network characteristics and CRAS is that more numerous and more interconnected social ties foster social and cultural aspects of communicative competence especially, thus protecting migrants’ against the negative effects of intercultural communication difficulties. To support this idea, it is important to show that L2 social network size and structure can predict CRAS above and beyond linguistic knowledge, indexed by self-reported language proficiency.

### Overall Intimacy in the L2 Social Network

The nature of the relation between strength of L2 social ties and L2 communication is unclear. Kim (2001) argues that while strong ties (especially marital relationships) are particularly important for cross-cultural adaptation, all ties (including weak ones) are sources of information helping migrants learn L2 cultural communication patterns. However, measures of the overall strength of migrants’ L2 social ties may be important for understanding acculturative stress in general, because such measures may serve as a proxy for the level of emotional support they can expect from their L2 network. A number of studies have established a positive relation between emotional support and adjustment among migrants (e.g., Crockett et al., 2007; Schneider and Ward, 2003). Therefore, it is important to control for this variable.

### Communication-Related Acculturative Stress (CRAS) vs. General Acculturative Stress (GAS)

To further support a case for the role of L2 social networks in promoting social and cultural aspects of communicative competence in the L2, it is important to show their association with CRAS above and beyond any association with general acculturative stress (GAS). To do so, CRAS scores will be residualized on GAS scores, thus eliminating shared variance between the two. To further investigate the specificity of the relation between L2 social network characteristics and CRAS, we will probe whether the association between L2 characteristics and communicative aspects of acculturative stress is stronger than with other aspects of acculturative stress. Past research has established a positive connection between social ties and mental health (Kawachi and Berkman, 2001), suggesting a possible negative relation between L2 network size and GAS. However, we would expect measures of L2 network interconnectedness, because of the fundamental role communication plays in establishing network interconnections, to be primarily related to communicative aspects over and above other aspects of acculturative stress. This test is also in line with our earlier proposal that it is important to examine the various facets of acculturative stress independently.

## Present Study

This study examines the specificity of the association between L2 social network characteristics and CRAS. In line with this goal, we focus on linguistically defined social ties instead of social ties in general. We have two research hypotheses:

H1: Greater L2 social network size (the number of members) and interconnectedness (inclusiveness and density within the L2 network) will be associated with lower CRAS residualized on GAS, after controlling for years in the new country, sex, acculturation orientations, self-reported language proficiency, average intimacy level in the L2 network.

The main idea underlying this hypothesis is that L2 social networks play an important role in fostering social and cultural aspects of communicative competence, and therefore in protecting migrants’ against intercultural communication difficulties. To further investigate the degree of specificity of the relation hypothesized above, namely that the relationship is specific to CRAS and not GAS, we formulate the following secondary hypothesis:

H2: The association between interconnectedness measures (inclusiveness and density) in the L2 social network and residualized CRAS will be stronger than the association between interconnectedness measures and GAS residualized on CRAS.

H2 is in fact almost exactly the obverse of H1 (namely that the residualization is the exact reverse of that specified in H1) and so can be coherently interpreted in light of the outcome of the analyses regarding H1.

## Materials and Methods

### Participants and Procedure

Multicultural students were recruited at an English-speaking university in Montreal, QC, Canada for a study on culture, identity, and language competence. The present sample included 100 participants (*M*_age_ = 24.18 years, SD_age_ = 4.20; 86 women) who were born outside of Canada and did not report English as their native language. Participants came from 46 different countries. On average, they had lived in Canada for 11.06 years (SD = 9.24). A student sample from an English-speaking university was chosen to ensure that all participants would have sufficient linguistic knowledge to be able to communicate and form new relationships in their L2 (English). Given their attendance at an English-speaking university located in a strongly English-speaking neighborhood of Montreal, we used English-speaking Canadians as the mainstream cultural reference group.

Participants were recruited using the local participant pool and received course credit as compensation for their time. The local Institutional Review Board approved the study. The study was administered online and took approximately 45 min to complete. After giving informed consent, participants provided demographic information and completed a number of measures.

### Measures

#### CRAS and GAS

The Riverside Acculturation Stress Inventory (RASI; Benet-Martínez and Haritatos, 2005) is a 15-item questionnaire assessing culture-related difficulties in different life domains (communication difficulties, work, intercultural relations, discrimination, and social isolation) on a 5-point rating scale. The mean score on three items assessing communication difficulties (e.g., ‘I often feel misunderstood or limited in daily situations because of my English skills’) constituted our measure of CRAS (Cronbach’s α = 0.65). The mean score on the remaining 12 items (e.g., ‘I feel the pressure that what “I” do is representative of my ethnic/cultural group’s abilities.’) constituted our measure of GAS (Cronbach’s α = 0.80). Total scores can range from 1 to 5. A recent psychometric study showed the RASI is a valid and reliable acculturative stress questionnaire (Miller et al., 2011).

#### Social Network Characteristics

Using an egocentric network survey, participants nominated up to 15 friends who are native English-speakers and with whom they typically interact in English. They rated their level of intimacy with each friend on a 4-point rating scale. They also indicated whether each pair of friends knew one another. Four indices were derived: L2 network size (number of L2 friends nominated); L2 intimacy (average intimacy rating across all friends nominated); L2 inclusiveness (number of non-isolated friends / total number of friends); and L2 density (number of existing links among nominated friends / number of possible links).

#### Acculturation Orientations

The Vancouver Index of Acculturation (VIA; Ryder et al., 2000) is a 20-item questionnaire that assesses orientations toward the mainstream (VIA-M subscale) and heritage (VIA-H subscale) cultural groups on a 9-point rating scale. Both subscales consist of 10 items with parallel wording. A sample item is: ‘I am comfortable working with typical English–Canadian people/people of the same heritage culture as myself.’ Total scores can range from 1 to 9. Past research has shown that the VIA is a valid and reliable acculturation orientation questionnaire (Ryder et al., 2000; Huynh et al., 2009). Internal consistency in this sample was good for both mainstream (Cronbach’s α = 0.82) and heritage (Cronbach’s α = 0.88) subscales. A Quebec version of the VIA distinguishes between English–Canadians and French–Canadians as possible mainstream groups. As noted earlier, English-speaking Canadians served as the mainstream reference group.

#### Self-Reported L2 Proficiency

Four in-house items assessed participants perceived ability to understand, speak, read, and write English on a 5-point rating scale. Total scores can range from 1 to 5. Internal consistency was very good (Cronbach’s α = 0.92).

### Analytic Approach

In line with our hypotheses, outcome variables were residualized: CRAS scores were residualized on GAS and vice-versa. We used multiple regression to test the study hypotheses. Variables were entered hierarchically to examine the unique predictive ability of social network size and structure. In terms of data preparation and screening, univariate outliers were winsorized to three median absolute deviations around the median (Leys et al., 2013). Two multivariate outliers were excluded from the analysis based on their robust Mahalanobis distance (Filzmoser et al., 2005) at a stringent level of *p* < 0.001, leaving 98 participants for the analysis. Unsurprisingly, density and inclusiveness were collinear and were therefore examined in separate models. We verified that statistical assumptions of the linear model were met through model diagnostics. Self-reported L2 proficiency suffered from serious range restriction (participants reported near perfect proficiency) and winsorizing the variable eliminated all residual variation. To retain some variation, we created two categories: participants with average L2 proficiency scores of 5 (ceiling L2 proficiency group) and participants with average L2 proficiency scores lower than 5 (non-ceiling L2 proficiency group). Finally, one observation was removed from analysis based on an overly large Cook’s distance (Fox and Weisberg, 2011) in regression diagnostics, leaving a total sample size of *N* = 97. All analyses were conducting using R version 3.1.2 (package igraph 0.7.1 for network analyses).

## Results

### Descriptive Results

**Table 1** shows the correlations of the continuous study variables. On average, participants reported low levels of communication-related (*M* = 2.00, SD = 0.96) and general (*M* = 2.56, SD = 0.76) acculturative stress, as measured by the RASI. Just over half the participants (52, or 54%) were in the ceiling L2 proficiency group, indicating native-like self-rated linguistic knowledge of English and 46 (46%) indicating less than native-like self-rated linguistic knowledge of English. Participants nominated 5.79 L2 friends on average (SD = 3.47) and their L2 social network was moderately inclusive (*M* = 0.53, SD = 0.39) and not very dense (*M* = 0.21, SD = 0.27). Participants reported a moderate level of intimacy with their L2 friends on average (*M* = 2.88, SD = 0.68). Participants’ acculturation orientations, measured by the VIA, were more positive toward their heritage group (*M* = 7.29, SD = 1.33) than toward the mainstream (English-speaking) cultural group (*M* = 6.82, SD = 1.23), but they were fairly positive in both cases.

**Table 1:** Zero-order intercorrelations of continuous study variables.

Variable		2	3	4	5	6	7	8	9
1.	CRAS	0.30**	-0.25*	-0.32**	-0.21*	-0.09	-0.14	-0.08	-0.33***
2.	GAS	—	0.02	-0.04	-0.01	-0.06	0.13	0.18^†^	-0.03
3.	L2 network size		—	0.44***	-0.05	-0.07	-0.01	-0.02	-0.02
4.	L2 inclusiveness			—	0.71***	-0.01	0.02	0.11	0.07
5.	L2 density				—	0.12	0.08	0.15	0.12
6.	L2 intimacy					—	0.33**	0.03	0.05
7.	VIA-M						—	0.18^†^	0.18^†^
8.	VIA-H							—	0.06
9.	Years in Canada								—

^†^*p* < 0.10; *p < 0.05; **p < 0.01; ***p < 0.001. CRAS, Communication-related acculturative stress; GAS, General acculturative stress; VIA-M, Vancouver Index of Acculturation – Mainstream subscale; VIA-H, Vancouver Index of Acculturation – Heritage subscale.

Zero-order correlations provide initial support for H1. L2 network size, *r* = –0.25, *p* = 0.01, 95% *CI* = [–0.43;–0.05], and both interconnectedness measures – L2 inclusiveness, *r* = –0.32, *p <* 0.001, 95% *CI* = [–0.49;0–013], and L2 density, *r* = –0.21, *p* = 0.04, 95% *CI* = [–0.39;–0.01] – are significantly correlated with CRAS. As initial support for H2, neither L2 inclusiveness, *r* = –0.04, *p* = 0.70, 95% *CI* = [–0.24;0.16], nor L2 density, *r* = –0.01, *p* = 0.92, 95% *CI* = [–0.21;0.19]. are associated with GAS scores in a statistically significant way.

### Hypothesis 1: Predicting Residualized Communication-Related Acculturative Stress (CRAS)

The left panel of **Table 2** presents results of the regression predicting CRAS (residualized on GAS), as measured by the RASI, using L2 network inclusiveness as a measure of interconnectedness. Longer time lived in Canada is associated with lower CRAS, [β (SE) = –0.27 (0.09), *p* = 0.004, 95% *CI* = [–0.44;–0.09]], and greater L2 proficiency – a categorical variable (ceiling versus non-ceiling self-reported L2 proficiency) – is associated with lower CRAS, β (SE) = –0.87 (0.16), *p* < 0.001, 95% *CI* = [–1.19;–0.55], indicating that, on average, CRAS scores for participants in the ceiling proficiency group are almost a full standard deviation lower than for participants in the non-ceiling group. Moreover, a simple Welch’s *t*-test revealed that CRAS scores in the ceiling proficiency group are significantly lower than in the non-ceiling proficiency group, *t′* = –6.54, *df* = 70.09, *p* < 0.001*)*. Also, being a male is associated with higher levels of CRAS, β (SE) = 0.49 (0.23), *p* = 0.03, 95% *CI* = [0.04;0.93]. Neither of the acculturation orientations, nor L2 intimacy, was significantly related to CRAS.

**Table 2:** Multiple regression of Communication-Related Acculturative Stress (CRAS) and General Acculturative Stress (GAS) with inclusiveness as a measure of interconnectedness.

	CRAS as outcome		GAS as outcome	
	Model 1 β (SE)	Model 2 β (SE)	Model 1 β (SE)	Model 2 β (SE)
Intercept	0.10 (0.60)	0.29 (0.60)	-1.72 (0.59)**	-1.73 (0.60)**
Sex (male)	0.36 (0.22)	0.44 (0.21)*	0.23 (0.20)	0.24 (0.21)
Years in Canada	-0.03 (0.01)**	-0.02 (0.01)**	0.01 (0.01)	0.01 (0.01)
VIA-M	0.04 (0.07)	0.04 (0.06)	0.17 (0.06)**	0.17 (0.06)*
VIA-H	-0.09 (0.06)	-0.07 (0.05)	0.07 (0.05)	0.07 (0.05)
L2 proficiency (ceiling group)	-0.86 (0.15)***	-0.79 (0.15)***	-0.26 (0.15)^†^	-0.26 (0.15)^†^
L2 intimacy	0.03 (0.11)	0.03 (0.11)	-0.13 (0.11)	-0.13 (0.11)
L2 network size		0.00 (0.02)	0.03 (0.02)	0.04 (0.02)
L2 inclusiveness		-0.69 (0.20)***		-0.40 (0.20)
*R^2^* total	0.386	0.473	0.143	0.143
Adjusted *R^2^*	0.345	0.425	0.075	0.065
*F* total	9.44 (6,90)***	9.85 (8,88)***	2.12 (7,89)*	1.84 (8,88)^†^
Δ*R*^2^		0.09		0.00

^†^*p* < 0.10, *p < 0.05, **p < 0.01, ***p < 0.001. *β* (SE), unstandardized regression coefficients (standard error).

In support of H1, introducing social network characteristics (L2 network size and L2 inclusiveness) into the model resulted in a statistically significant 9% increase in explained variance, *F*(2,88) = 7.18, *p* = 0.001) and in higher inclusiveness being associated with lower CRAS, β (SE) = –0.30 (0.09), *p* < 0.001, 95% *CI* = [–0.46;–0.13]. Contrary to H1, however, a larger social network alone did not predict lower CRAS in the final model, β (SE) = 0.02 (0.09), *p* = 0.85, 95% *CI* = [–0.16;0.19]. Given the statistically significant zero-order correlation between L2 network size and CRAS (*r* = –0.25, *p* = 0.01), we examined the role of L2 network size further. Supplementary analyses revealed that – after controlling for sex, age, acculturation orientations, and L2 intimacy – L2 network size was a significant predictor of CRAS, β (SE) = –0.26 (0.09), *p* = 0.004, 95% *CI* = [–0.44;–0.08]). Introducing L2 proficiency and L2 inclusiveness into the model eliminated the effect of L2 network size, reducing the strength of the association from β = –0.26 to β = 0.02. This indicates that the variable L2 network size shares most of its variance with L2 proficiency and L2 inclusiveness.

The left panel of **Table 3** presents results of the regression predicting CRAS using density as a measure of interconnectedness. In this model, also supporting H1, the introduction of social network characteristics (L2 network size and L2 density) resulted in a statistically significant 6% increase in explained variance, *F*(2,88) = 4.26, *p* = 0.02. In line with H1, higher density was also associated with lower CRAS, β (SE) = –0.19 (0.08), *p* = 0.01, 95% *CI* = [–0.35;–0.04]. Comparing association strengths for inclusiveness and density with CRAS shows that inclusiveness is a better predictor of CRAS than density, β = –0.30 vs. β = –0.19. In addition, inclusiveness accounts for more unique variance in CRAS than density, Δ*R*^2^ = 0.07 with inclusiveness entered at the last step, versus Δ*R*^2^ = 0.04 for entering density at the last step.

**Table 3:** Multiple regression of CRAS and GAS with density as a measure of interconnectedness.

	CRAS as outcome		GAS as outcome	
	Model 1 β (SE)	Model 2 β (SE)	Model 1 β (SE)	Model 2 β (SE)
Intercept	0.10 (0.60)	0.29 (0.62)	-1.72 (0.59)**	-1.71 (0.60)*
Sex (male)	0.36 (0.22)	0.38 (0.21)	0.23 (0.20)	0.23 (0.21)
Years in Canada	-0.03 (0.01)**	-0.03 (0.01)**	0.01 (0.01)	0.01 (0.01)
VIA-M	0.04 (0.07)	0.04 (0.07)	0.17 (0.06)**	0.17 (0.06)*
VIA-H	-0.09 (0.06)	-0.07 (0.05)	0.07 (0.05)^†^	0.07 (0.05)
L2 proficiency (ceiling)	-0.86 (0.15)***	-0.79 (0.15)***	-0.26 (0.15)^†^	-0.26 (0.15)^†^
L2 intimacy	0.03 (0.11)	0.05 (0.11)	-0.13 (0.11)	-0.13 (0.11)
L2 network size		-0.03 (0.02)	0.03 (0.02)	0.03 (0.02)
L2 density		-0.67 (0.27)*		0.09 (0.26)
*R^2^* total	0.386	0.441	0.143	0.144
Adjusted *R^2^*	0.345	0.390	0.075	0.066
*F* total	9.44 (6,90)***	8.66 (8,88)***	2.12 (7,89)*	1.85 (8,88)^†^
Δ*R*^2^		0.06		0.00

^†^*p* < 0.10, *p < 0.05, **p < 0.01, ***p < 0.001. *β* (SE), unstandardized regression coefficients (standard error).

### Hypothesis 2: Predicting Residualized GAS

The right panel of **Table 2** presents results of the regression predicting GAS (residualized on CRAS), as measured by the RASI, using L2 inclusiveness as a measure of interconnectedness. More positive mainstream acculturation orientation, as measured by the VIA-M, are associated with higher levels of GAS, β (SE) = 0.30 (0.11), *p* = 0.01, 95% *CI* = [0.07;0.52]. None of the other predictors was significantly related to GAS. Supporting H2, the confidence interval of the inclusiveness coefficient in the regression of CRAS scores (βinclusiveness-CRAS *CI* = [–0.46;–0.13], reported in the previous section) did not include the inclusiveness coefficient in the present regression predicting GAS (βinclusiveness-GAS = –0.02). Further supporting the possibility that L2 interconnectedness is associated primarily with communicative aspects of acculturative stress, L2 inclusiveness did not predict residualized GAS scores significantly, β (SE) = –0.02 (0.11), *p* = 0.85, 95% *CI* = [–0.23;0.19]. In addition, introducing inclusiveness in the model did not explain any additional variance, *F*(1,88) = 0.04, *p* = 0.85, compared to 7% additional explained variance when introducing this variable in the regression of CRAS scores. Collectively, these results fully support our second hypothesis for inclusiveness.

The right panel of **Table 3** presents results of the regression predicting residualized GAS using L2 network density as a measure of interconnectedness. As in the case of inclusiveness, introducing L2 density did not explain any additional variance in GAS scores, *F*(1,88) = 0.12, *p* = 0.73, compared to 4% additional explained variance when introducing this variable in the regression of CRAS scores. In further support of H2, the confidence interval of the density coefficient in the regression of CRAS scores (βdensity-CRAS *CI* = [–0.35;–0.04], reported in the previous section) did not include the density coefficient in the present regression predicting GAS scores (βdensity-GAS = 0.03). Accordingly, the coefficient for L2 density was not statistically significant, β (SE) = 0.03 (0.09), *p* = 0.73, 95% *CI* = [–0.16;0.22]. These results show that interconnectedness measures are associated with CRAS, above and beyond any association with GAS, and that this relation is stronger than between interconnectedness and residualized GAS. Taken together, the results support the idea that L2 social network interconnectedness is associated with communicative aspects of acculturative stress but not with other aspects.

## Discussion

For migrants, the process of adapting to a new cultural environment occurs largely through L2-mediated communication with members of the new mainstream cultural group (Kim, 2001). In this study, we focused on migrants’ subjective stress reaction in response to chronic difficulties in this type of intercultural communication. Our first hypothesis that migrants’ L2 social network size and interconnectedness would predict CRAS was mostly supported. Larger L2 network size, higher L2 inclusiveness, and higher L2 density were all statistically significantly associated with lower CRAS scores. In addition, both interconnectedness measures uniquely accounted for a significant proportion of variance in the outcome variable after controlling for important covariates. These results support the idea that more interconnected L2 social networks can to some extent protect migrants against the negative psychological effects of intercultural communication difficulties.

However, contrary to our hypothesis, L2 network size did not uniquely predict CRAS. A closer look at the results indicated that the positive and significant relation between these variables disappeared once self-reported L2 proficiency was entered into the model. A possible explanation for this finding is that L2 proficiency mediates the relation between L2 network size and CRAS. Having more L2 friends means more occasions to use the L2. This may help migrants develop their linguistic knowledge of the L2, thus resulting in higher self-reported L2 proficiency scores. In turn, better L2 proficiency facilitates L2 communication in general. This is consistent with past research showing that greater L2 use is associated with greater L2 proficiency (Gatbonton and Trofimovich, 2008).

The finding that interconnectedness, but not L2 network size, accounted for unique variance in CRAS underscores the notion that the structure of L2 social networks matters. This idea, widely accepted in the social network literature (Butts, 2008; Borgatti et al., 2009), has received limited empirical attention in cross-cultural psychology research. Studies using social network variables to predict L2 or acculturation related phenomena typically focus on social network size rather than on structural variables (e.g., Cenoz and Valencia, 1993; García et al., 2002; Wiklund, 2002; Hendrickson et al., 2011). More recently, researchers have started taking structural aspects of social networks into consideration (e.g., Mok et al., 2007; Gallagher, 2013). The present study contributes to this limited body of work by showing that while the size of migrants’ L2 friendship networks is not sufficient to predict CRAS, the configuration of these friendship networks has unique predictive ability. More broadly, these results suggest that not all types of social contact and language use may equally facilitate migrants’ communicative competence and relieve associated stress. An interesting direction for future research therefore might be to identify the characteristics of social contact and L2 use situations that might moderate this relation.

Similarly, future research should examine the role of L2 social networks in CRAS in other cultural environments. Montreal is a very specific setting, characterized by two mainstream cultural groups: Francophone Canadians, and Anglophone Canadians. Although English is the dominant local language in the neighborhood where the study was conducted, Francophone Canadians represent the overall numerical majority in Montreal and in the rest of the province. This complexity in the cultural and linguistic composition of the wider study context renders a clear definition of “L2 social networks” difficult. Thus, it would be interesting to extend the current investigation to other cultural settings where the immigrants’ L2 community is also the only mainstream cultural group (e.g., Turkish immigrants and Germans in Germany)

As mentioned in the Section “Introduction,” a potential mechanism underlying the relation between interconnectedness of the L2 social network and CRAS is that more interconnected networks facilitate observational learning of normative language forms and communicative competence by increasing the likelihood of triadic interactions involving two or more native speakers of the L2, thus providing greater access to unmodified communication practices. Thus, interconnected L2 social networks may foster the learning of both cultural representations and L2 communication practices, which are closely intertwined. In future research, it would be important to further unpack how exactly greater interconnectedness helps learning. Cultural schemata for social interactions (Nishida, 1999) may provide a useful starting point for such an exploration. Cultural schemata for social interactions are, “cognitive structures that contain knowledge for face-to-face interactions in one’s cultural environment,” (Nishida, 2005, p. 403) and that guide communication in this environment. Like other cultural schemata (Casson, 1983; D’Andrade, 1992), they emerge out of repeated engagement with particular cultural contexts and become more organized, abstract, and compact through repeated use. In the process, they increasingly guide people’s negotiation through their social environment. Cultural schemata for social interactions organize knowledge of cultural norms and preferences and as well as linguistic knowledge. In line with the above perspective on observational learning, more interconnected L2 social networks may facilitate the acquisition and automatization of cultural schemata for social interactions, which may in turn lead to more successful L2 communication, thus acting as a mediator.

Perhaps this greater access to unmodified natural cultural representations and L2 communication practices serves as a mechanism underlying the relation between interconnectedness and CRAS and thus also helps to explain the finding that L2 network inclusiveness was a stronger predictor than density. These two indices measure different stages in the formation of an interconnected social network; low inclusiveness entails low density, whereas high inclusiveness (even at a ceiling value of 1) can be associated with either low or high density. Perhaps early stages of social tie formation lead more to greater inclusiveness than to greater internal density (that is, people first get to know more individuals who are themselves somewhat connected to each other (a few classmates, workmates, immediate neighbors, etc.) and this translates into opportunities for L2-mediated interactions with two or more members of the mainstream group (since these network members know one another). This in turn potentially results in greater observation learning. Once an inclusive mainstream network is achieved, however, increased interconnectedness within the network might accrue little additional advantage.

The results presented here also supported our primary and secondary hypotheses when viewed together that greater interconnectedness in the L2 social network would be associated primarily with communicative aspects of acculturative stress (residualized on GAS) but not GAS (residualized on communicative acculturative stress), reinforcing the idea that the role of L2 interconnectedness is specific to L2 communication aspects of stress and is not simply a stress reliever in general. Nevertheless, it would be important in future research to study the role of L2 network variables in predicting other facets of acculturative stress separately. For example, given the hypothesized role of sustained relationships with members of the mainstream group in learning new cultural schema and minimizing intercultural strain, it would be interesting to examine the ability of these L2 network variables to predict acculturative stress arising from strained intercultural relations.

The ability of L2 interconnected to predict CRAS was in line with our hypothesis, but the finding that L2 network size did not predict GAS at all was somewhat surprising, given the existing literature on the salutary effects of social ties on mental health. One possible explanation of this null effect is the fact that we did not measure network size in general but the L2 network size specifically. Participants with few L2 friends may have a large social network in their L1 on which they rely for support and help meeting non-language-related acculturative hurdles. Another possibility lies in the fact that social ties have costs associated with them, expressed as social obligations and expectations of reciprocity (Kawachi and Berkman, 2001). Nevertheless, the differential pattern of results in predicting communication-related versus GAS lends some support to Rudmin’s (2009, p. 116) argument that acculturative stress may be a, “catch-all concept for every kind of problem that minorities might encounter,” and is in line with past research documenting the multifaceted relation between various aspects of acculturative stress and bicultural identity integration (Benet-Martínez and Haritatos, 2005). We believe that research on cross-cultural adaptation would benefit from disentangling the various facets of acculturative stress and this study represents one step in that direction by showing that L2 social network interconnectedness is specifically related to CRAS and not to other facets of acculturative stress.

Despite this study’s contribution, we want to discuss some limitations. First, reliance on a student sample, and the fact that most of the sample was female, limits the generalizability of the results and introduces potential pressures on social interactions and the form of social relationships that are captured by the L2 network. By attending university in their L2, students are more forced to pursue intercultural communication everyday and may have more opportunities to form relationships with native speakers of their L2 than community members whose days are more tightly structured by work obligations. Related to this, the L2 network was defined in “friendship” terms, which may differ across sexes. Also, friendship is a more central concern for young adults – the typical student population – than for people with family responsibilities. It is quite possible that the L2 network would still play a role in alleviating CRAS in a community sample, but the network instrument would have to be adapted to reflect the lived experience of migrants who work and take care of a family. It could, for example, include work relations and acquaintances.

A second limitation concerns the range restriction in English proficiency we observed in our sample. Participants reported very high levels of English proficiency and relatively low levels of acculturative stress on average, indicating good functioning in the new cultural environment overall. It is important to note, however, that we observed the same pattern of results when excluding participants in the “ceiling” L2 proficiency group from the analyses (the *p*-value for density decreased to 0.06 in the prediction of CRAS, but this likely due to a loss of power resulting from shrinking our sample size to 46 participants). In a way, this limitation in terms of range restriction, which raises questions about the generalizability of the results to people facing more difficulties with cross-cultural adaptation, is closely tied to participants being students. By definition, attending daily classes at an English-speaking university assumes a good level of English proficiency. Nevertheless, even such a sample would be expected to exhibit variation in L2 fluency and in more subtle sociolinguistic aspects of language proficiency. Our self-report measure did not allow us to detect this variation, but in future research it would be useful to use objective language measures assessing various aspects of linguistic knowledge.

A third limitation concerns the cross-sectional and correlational nature of the study, which prevents any inferences regarding the causality or temporal order in the relation between L2 interconnectedness and communication-related stress. For example, as an alternative to the observational learning mechanisms discussed earlier, people who experience more communication-related stress may feel more comfortable with L1 friends and not seek out or maintain L2 friendships. In the future, longitudinal studies could examine whether increases in L2 network size and interconnectedness prospectively predict decreased communication-related stress or whether the reverse temporal direction is supported. As a matter of fact, mutually reinforcing bidirectional effects – whereby more numerous and more interconnected L2 friends buffer against communication-related stress, which in turn facilitates L2 friendships formation – are likely. Better understanding these mechanisms could have important implications for interventions aimed at facilitating L2 learning and cross-cultural adaptation.

In spite of these limitations, the present study supports the idea that more interconnected L2 social networks can be protective against the negative psychological effects of L2 communication difficulties. In doing so, it shows that the structure of migrants’ L2 network matters for language related outcomes. This study also contributes to the emerging body of work that attempts to integrate cultural/cross-cultural research on acculturation and applied linguistics research on communication and SLA. Of relevance to both strands of research, the present work provides support for the view that adapting to a new cultural environment and learning its normative communication practices are not only intertwined but also fundamentally social phenomena occurring through social interactions and relationships.

## Author Contributions

MD contributed to the design of the study, to data collection and analysis, and to data interpretation, and she drafted and contributed to revising the manuscript. RS contributed to data collection, to data interpretation, and to revising the manuscript. NS contributed to the design of the study, to data interpretation, and to revising the manuscript. AR contributed to the design of the study, to data interpretation, and to revising the manuscript. All authors approve the final version of this manuscript and agree to be accountable for all aspects of the work in ensuring that questions related to the accuracy or integrity of any part of the work are appropriately investigated and resolved.

## Conflict of Interest Statement

The authors declare that the research was conducted in the absence of any commercial or financial relationships that could be construed as a potential conflict of interest.
